# TRPV1 Blocker, Peptide HCRG21 from Sea Anemone *Heteractis magnifica*, Exhibits Effectiveness in Psoriasis and Dermatitis in In Vivo Models

**DOI:** 10.3390/ijms262110644

**Published:** 2025-10-31

**Authors:** Anna Klimovich, Aleksandra Kvetkina, Yulia Deryavko, Nadezhda Priymenko, Daria Popkova, Evgenia Bystritskaya, Marina Isaeva, Irina Gladkikh, Oksana Sintsova, Elena Leychenko

**Affiliations:** 1 G.B. Elyakov Pacific Institute of Bioorganic Chemistry, Far Eastern Branch, Russian Academy of Sciences, 690022 Vladivostok, Russia; annaklim_1991@mail.ru (A.K.); kvetkinaan@gmail.com (A.K.); yliya77ya@mail.ru (Y.D.); leopoltik07@mail.ru (N.P.); daria.vladipo@yandex.ru (D.P.); ep.bystritskaya@yandex.ru (E.B.); issaeva@gmail.com (M.I.); irinagladkikh@gmail.com (I.G.); sintsova0@gmail.com (O.S.); 2 Advance Engineering School “Institute of Biotechnology, Bioengineering and Food Systems”, Far Eastern Federal University, 690920 Vladivostok, Russia

**Keywords:** Kunitz-type peptide, TRPV1 channel, skin inflammation, psoriasis, allergic contact dermatitis, cytokines, imiquimod-induced model

## Abstract

Psoriasis and allergic contact dermatitis (ACD) are the most common chronic inflammatory diseases, which are accompanied by epithelial alterations and a T cell-mediated immunopathology. In this study, we investigated the anti-ACD and anti-psoriasis effects of sea anemone *Heteractis magnifica* peptide HCRG21, a blocker of the TRPV1 channel, in 2,4-dinitrofluorobenzene (DNFB)- and imiquimod (IMQ)-induced mouse models, respectively. We found that topical application of 0.005–0.1% HCRG21 gels normalized hematological and immunological blood parameters in mice, significantly reduced the severity of ACD- and psoriasiform-like skin lesions, and increased the rate of tissue repair. The use of 0.005 and 0.05% HCRG21 gels decreased the production of IL-23-A and macrophage-derived chemokine (MDC) proteins in blood plasma, reduced the expression of *Tnf*, *Il1β*, *Il6*, *Il23a*, and *Il17a* genes, but increased the levels of the *Il10* gene in scabs and/or blood of IMQ-treated mice. On the other hand, topical application of 0.05 and 0.1% HCRG21 reduced the expression of *Il6* and *Il23a* in the DNFB-treated mice’s blood and it had no significant effects on TNF-α and IL-1β production. Thus, HCRG21 has the potential to be a treatment for psoriasis and dermatitis due to its potent anti-inflammatory properties. This effect is achieved by reducing pro-inflammatory cytokines associated with TRPV1 and normalizing immune cell levels in the bloodstream. This, in turn, leads to a decrease in clinical symptoms and an improvement in skin healing.

## 1. Introduction

The skin is a complex barrier organ, playing a vital role in maintaining homeostasis, and protecting the body against external influences and infections that trigger various inflammatory processes, such as immune responses, autoimmunity, and allergy [1]. Psoriasis and allergic contact dermatitis (ACD) are the most common chronic inflammatory disorders of the skin, with pathogeneses that are complex and multifactorial. These diseases are characterized by immunological dysfunction, which is accompanied by pain, itching, long non-healing wounds, and deterioration of barrier functions and skin quality.

ACD is a classic example of a type IV hypersensitivity reaction. At the first stage, receptor proteins of dendritic cells sensitize external small molecules known as haptens or contact allergens and present the hapten–protein complex to T cells, which in turn form an expanded population of allergen-specific memory T cells [2]. Reexposure to hapten initiates transmigration of the expanded memory T cells to the dermis and epidermis, which interact with antigen-presenting cells followed by the triggering an inflammatory immune response through pro-inflammatory cytokine (TNF-α, IL-1β, IL-6, and IL-23-A) production, resulting in a clinical manifestation of ACD at the sites of hapten challenge [3,4].

Psoriasis is clinically characterized by well-defined erythematous papules or plaques covered with silvery-white scales [5]. Although the etiology of psoriasis is not completely understood, it is considered to be caused by genetic and environmental factors that trigger the production of pro-inflammatory cytokines and chemokines by skin cells, resulting in hyperproliferation and abnormal differentiation of keratinocytes and massive infiltration of inflammatory immune cells to the skin tissue [6,7]. There are many types of cells, including keratinocytes, T cells, dendritic cells, neutrophils, and macrophages, involved in psoriasis development [8]. Similar to ACD, psoriasis is caused primarily by TNF-α, IL-1β, and IL-6 produced by keratinocytes, which stimulate plasmacytoid dendritic cells to produce interferon-α (IFN-α) followed by its stimulation of IL-12 and IL-23 release by dendritic cells nearby lymph nodes. These cytokines then activate circulating T helper cells, which subsequently move to the skin and produce IFN-γ, IL-17A, IL-17F, and IL-22 cytokines, resulting in inflammation and uncontrolled hyperproliferation of keratinocytes [5,9].

Current treatments for psoriasis and contact dermatitis often face limitations such as incomplete responses, adverse effects, poor patient compliance, and difficulties in identifying triggers or optimal therapy. Topical corticosteroids, the first-line treatment for both conditions, are effective but are associated with significant adverse effects such as skin thinning, making long-term use problematic [10,11]. These issues highlight the urgent need for new, targeted, and personalized therapeutic approaches that are both effective and safer for chronic use.

At present, much attention has been paid to the involvement of ion channels in the pathogenesis of skin diseases, in particular, ACD and psoriasis [12]. Previous studies have shown that the Transient Receptor Potential Vanilloid 1 (TRPV1) channel is closely related to the development of various chronic inflammatory skin diseases, such as psoriasis [13], dermatitis [14], rosacea [15], herpes zoster [16], and prurigo nodularis [17]. This ion channel is involved in the regulation of a number of physiological processes and pathological conditions such as pain, thermoregulation, inflammation, neuropathies, asthma, itching, and cardiovascular disorders [18,19,20]. In the skin, TRPV1 is present on the membranes of keratinocytes, mast cells, Langerhans cells, sebocytes, sweat gland cells, and hair follicles, and it plays an important role in skin inflammation, pain, and itching, contributing significantly to the pathogenesis of dermatitis and psoriasis [21,22]. The overexpression of TRPV1 in pruritic skin correlates positively with the intensity of psoriasis itching. Indeed, TRPV1 gene knockout has been shown to result in a significant decrease in the expression levels of inflammatory cytokines IL-1β, IL-6, and IL-23-A in the IMQ-induced murine model of psoriasiform dermatitis [23]. Suppression of the *Il23a* gene prevents the differentiation of T-helper 17 cells and their production of IL-17. In addition, TRPV1 activation stimulates the production of IL-23-A by dendritic cells of the dermis, which in turn triggers the formation of inflammatory edema [24]. Therefore, the TRPV1 channel is considered as a therapeutic target in the treatment of various inflammatory skin diseases, while the search for TRPV1 blockers possessing additional anti-inflammatory effects is a prospective task.

The Kunitz peptide HCRG21 (56 aa, 6228 Da, UniProt ID: P0DL86), found in the sea anemone *H. magnifica* during transcriptome profiling, has demonstrated attractive biological effects in different in vivo models, acting as a potent inhibitor of the TRPV1 ion channel [25,26,27]. HCRG21 was shown to induce prolonged analgesic effects in a hot plate test, as well as pronounced anti-inflammatory activity inhibiting carrageenan-induced paw edema by reducing TNF-α production to a control level [26,27]. Therefore, this molecule has powerful pharmacological potential for the treatment of the conditions where the activity of the TRPV1 ion channel must be temporarily reduced. Here, for the first time, the peptide TRPV1 blocker, HCRG21, was used to treat chronic inflammatory skin conditions, psoriasis, and ACD using IMQ- and DNFB-induced CD-1 mouse models, respectively. This work examined the main molecular markers of inflammation and revealed a positive effect of the peptide on the course of treatment and key cytokine expression levels when topical gels containing HCRG21 were applied.

## 2. Results

### 2.1. The Efficacy of HCRG21 in Allergic Contact Dermatitis

#### 2.1.1. HCRG21 Reduces DNFB-Induced ACD-like Lesions in Mice

**Figure 1:**
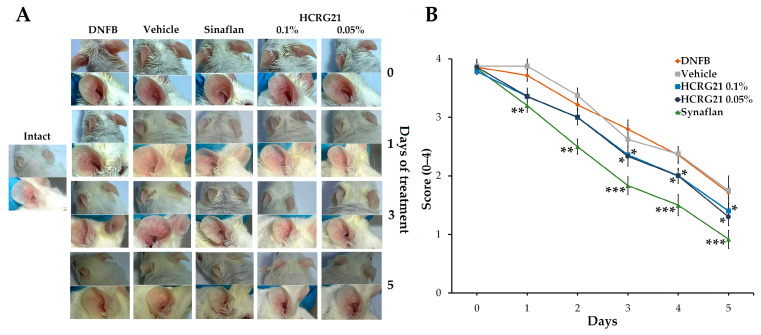
Effects of HCRG21 gels on DNFB-induced erythema. (**A**) Phenotypic observation of the ear skin in the experimental mice groups. (**B**) Erythema scores of ear dermatitis were evaluated daily. Intact—healthy mice, DNFB—mice treated with DNFB, Vehicle—mice treated with DNFB and gel without HCRG21, Sinaflan—mice treated with DNFB and Sinaflan, HCRG21 0.1%—mice treated with DNFB and HCRG21 0.1% gel, HCRG21 0.05%—mice treated with DNFB and HCRG21 0.05%. The data are shown as mean ± SEM (*n* = 7); * *p* < 0.05, ** *p* < 0.01, and *** *p* < 0.001 indicate significant differences from the DNFB group according to one-way ANOVA/Tukey’s test.

To evaluate the therapeutic effects of HCRG21 on skin inflammation, we used a DNFB-induced mouse ACD-like model. Application of DNFB on the ear skin on the 6th and 7th days resulted in redness and hardening of the inflamed skin area, which was accompanied by hyperemia and formation of surface hemorrhagic edema. As shown in Figure 1A, gels containing 0.05 and 0.1% HCRG21 significantly alleviated scales, erythema, and the thickness of epidermis compared to the intact group starting from the 3rd day of the treatment. The score decreased in both HCRG21 groups (*p* < 0.05) and Sinaflan (*p* < 0.001) on the 5th day compared to those in the DNFB group (Figure 1B). Nevertheless, Sinaflan more effectively reduced clinical manifestations of ACD than HCRG21 (*p* < 0.05).

#### 2.1.2. HCRG21 Normalizes Hematological Parameters in DNFB-Induced ACD-like Model

To determine the influence of 0.1 and 0.05% HCRG21 on whole blood cell content, a clinical blood analysis was carried out. According to the results, levels of total leucocytes (WBC) and platelets (PLT) in the DNFB group were markedly elevated by 52.5 and 57.1%, respectively (Table 1), compared to the intact group, which are the clinical signs of ACD. Conversely, in both HCRG21-treated groups, the levels of WBC and PLT were significantly alleviated by 14.8 and 10.7%, respectively, and by 20.1% in the blood compared to the values in DNFB-treated mice, while the levels of lymphocytes, monocytes, eosinophils, and basophils were within the range of normal values. At the same time, treatment of animals with the commercial ointment Sinaflan brought the total leukocyte level to normal and promoted a decrease in lymphocytes, monocytes, and basophils. In addition, the levels of PLT and thrombocytocrit in this group were higher than in the other groups, which is probably a consequence of one of the side effects of glucocorticosteroids—stimulation of thrombocytosis [28]. The levels of erythrocytes, hemoglobin, and hematocrit did not differ significantly in any of the experimental groups.

#### 2.1.3. HCRG21 Suppresses the Production of Cytokines in DNFB-Induced ACD-like Skin Lesions

To clarify the molecular mechanism of the anti-ACD effect of HCRG21, the expression levels of inflammatory cytokines, including IL-1β, IL-6, IL-23-A, and TNF-α, were estimated using qPCR.

**Figure 2:**
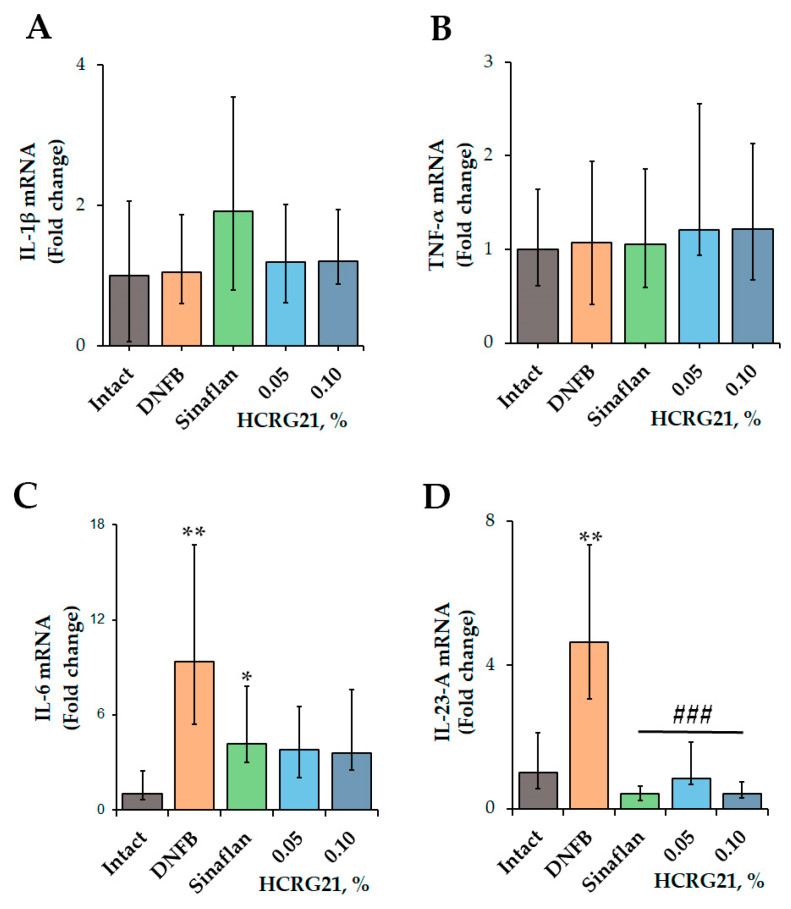
Effect of HCRG21 and Sinaflan on IL-1β (**A**), TNF-α (**B**), IL-6 (**C**), and IL-23-A (**D**) gene expression levels in the blood of mice treated and untreated with DNFB. Gels with 0.05 and 0.1% HCRG21 and Sinaflan (0.025%) were applied 24 h after the last DNFB application. Animal groups: Intact—healthy mice; DNFB—DNFB-treated mice; Sinaflan—DNFB and Sinaflan-treated mice (positive control); 0.05—mice, treated by DNFB and hydrogel containing 0.05% HCRG21; 0.1—mice treated by DNFB and hydrogel containing 0.1% HCRG21. Results were normalized to β-actin gene expression and presented as mean ± SEM (*n* = 7); * *p* < 0.05, and ** *p* < 0.01 indicate significant differences from the intact group, and ^###^ *p* < 0.001 indicate significant differences from the DNFB-treated group according to one-way ANOVA/Dunnett’s multiple comparisons test.

According to the qPCR results (Figure 2), application of DNFB resulted in an increase in the levels of IL-6 and IL-23-A genes by 89.3 and 78.4%, respectively, while the values of *Il1β* and *Tnf* were comparable with the levels of the intact group. The commercial ointment Sinaflan decreased the levels of both *Il6* and *Il23a* by 55.1 and 91.1%, respectively, but it increased the level of Il1β by 18.1% compared with the DNFB group values. Both HCRG21 0.05 and 0.1% gels significantly suppressed *Il23a* levels by 82 and 91.1%, respectively, in contrast to the DNFB group values, which were comparable with the Sinaflan effect. Notably, HCRG21 gels also inhibited IL-6 mRNA levels, but their values were insignificant, and they did not affect the IL-1β and TNF-α gene expression levels.

#### 2.1.4. HCRG21 Facilitates the Correction of Pathomorphological Changes in DNFB-Induced ACD-like Skin

**Figure 3:**
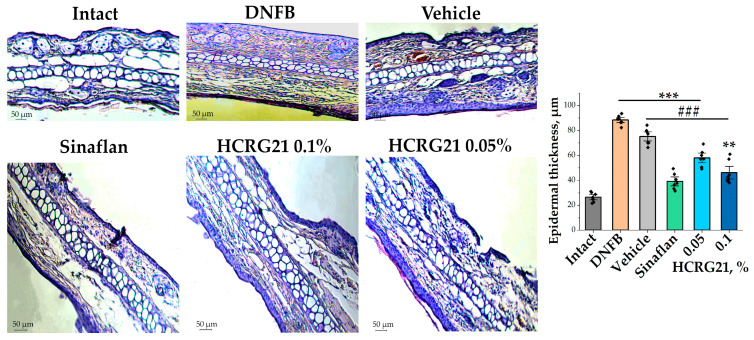
Histological sections of mice auricles at 20× magnification and changes in epidermal thickness in each group. Intact—healthy animals; DNFB—DNFB-treated animals; Sinaflan—DNFB and Sinaflan-treated animals; Vechicle—animals treated with DNFB and hydrogel without HCRG21; HCRG21 0.1%—animals treated with DNFB and gel containing 0.1% HCRG21; HCRG21 0.05%—animals treated with DNFB and gel containing 0.05% HCRG21. Tissue staining was performed with hematoxylin and eosin. The data are shown as mean ± SEM (*n* = 7); ** *p* < 0.01 and *** *p* < 0.001 indicate significant differences from the intact group and ^###^ *p* < 0.001 indicates significant differences from the DNFB group according to one-way ANOVA/Dunnett’s multiple comparisons test.

To estimate the effects of HCRG21 gels on morphological changes during ACD-like ear lesions, the tissues of the experimental mice were studied. Analysis of histological fragments of ears stained with hematoxylin and eosin allowed us to identify an abundance of blood vessels, often dilated and vascularized, with extensive hyperkeratosis of the epidermis 3.3 times, as well as epidermal and dermal hyperplasia in the DNFB group in contrast to the intact group (Figure 3). At the same time, significantly reduced hyperkeratosis and hyperplasia and levelled out pathological changes in the intradermal vessels were observed in histological sections of ears treated with Sinaflan and gels containing 0.1% and 0.05% HCRG21 (Figure 3). As seen in Figure 3, Sinaflan significantly reduced the epidermal thickness of mice ear auricles by 2.2 times compared to that in the DNFB-treated group. It is interesting to note that gels containing 0.1% and 0.05% HCRG21 had close therapeutic effects, significantly reducing epidermal thickness by 2 and 1.5 times, respectively, and other pathomorphological manifestations of erythema, as well as improving hematological and immunological counts.

### 2.2. The Efficacy of HCRG21 in Psoriasis-like Disease

#### 2.2.1. HCRG21 Alleviates IMQ-Induced Psoriasis-like Skin Lesions in Mice

**Figure 4:**
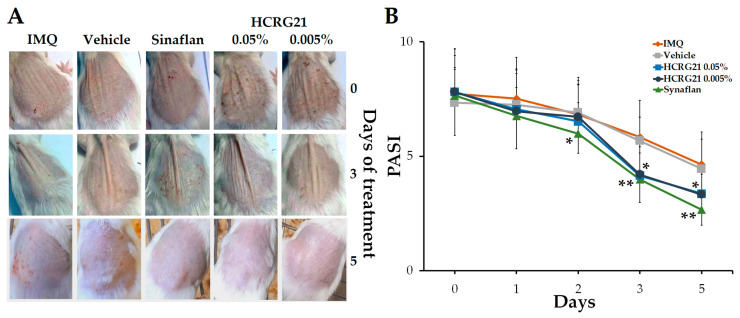
Effects of HCRG21 gels on IMQ-induced erythema. (**A**) Phenotypic observation of the neck skin in the experimental groups. (**B**) Daily scored PASI index (visual assessment of psoriasis severity: hyperkeratosis, desquamation, and hyperemia) of the psoriasiform skin. IMQ—mice treated with IMQ; Vehicle—mice treated with IMQ and gel without HCRG21, Sinaflan—mice treated with IMQ and Sinaflan, HCRG21 0.005%—mice treated with IMQ and HCRG21 0.005% gel, HCRG21 0.05%—mice treated with IMQ and HCRG21 0.05%. The data are shown as mean ± SEM (*n* = 7); * *p* < 0.05 and ** *p* < 0.01 indicate significant differences from the IMQ group according to one-way ANOVA/Tukey’s test.

To estimate the effect of HCRG21 on psoriasis-like skin, the IMQ-induced mouse model was used. After topically applying IMQ for 30 consecutive days, we noticed that the clinical manifestations of psoriasis were less severe in the HCRG21 groups and Sinaflan compared to the IMQ group. Application of IMQ resulted in hyperkeratosis with increased epidermis thickness, scaling, and scaly epithelium and hyperemia (Figure 4A). The PASI index calculated before the treatment averaged 7.5–8 points in all experimental groups (Figure 4B). Gels containing 0.05% and 0.005% HCRG21 and Sinaflan showed therapeutic effects after the third application and continued until the end of the experiment. The PASI scores in these groups significantly reduced to 4 points (*p* < 0.01) on the 3rd day and reached the minimum values on the 5th day of the experiment, while those of the IMQ group fluctuated around 5 points. As shown in Figure 4A, HCRG21 alleviated the severity of clinical manifestations of psoriatic skin lesions, which were evidenced by a reduction in the severity of hyperkeratosis, desquamation, and hyperemia, as well as an increase in the rate of epidermis recovery.

#### 2.2.2. HCRG21 Normalizes Hematological Blood Parameters in IMQ-Induced Psoriasis-like Model

Hematological analysis of IMQ-induced mouse blood allowed us to identify the changes in blood cell contents, which are associated with the development of psoriasis. A significant 3-fold increase in the total leukocyte, accompanied by pathological shifts in the leukocyte formula (neutrophils, eosinophils, and monocytes) was observed in the IMQ group of mice in contrast to the intact group (Table 2). Moreover, the IMQ group demonstrated a significant decrease in erythrocyte content and hemoglobin level, as well as an increase in platelet and thrombocytocrit values by 18.2 and 28.3%, respectively. In animals treated with 0.05% and 0.005% HCRG21 gels and Sinaflan, the hematological parameters were significantly decreased compared to the IMQ group. A five-day treatment course with 0.005% and 0.05% HCRG21 gels resulted in a reduction in whole leukocyte levels by 22.8 and 17.3%, respectively, in comparison with the IMQ group; in particular, the eosinophil and basophil contents were significantly diminished, and the lymphocyte and neutrophil ratio was normalized. Furthermore, the groups treated with HCRG21 gels exhibited decreased platelet levels and slightly increased erythrocyte and hemoglobin contents. Notably, the hematological parameters of the group treated with the Sinaflan ointment did not differ significantly from those of the HCRG21 groups, with the exception of platelet levels, which remained elevated in this group.

#### 2.2.3. HCRG21 Suppresses the Production of Cytokines in IMQ-Induced Psoriasis-like Skin Lesions and Blood

**Figure 5:**
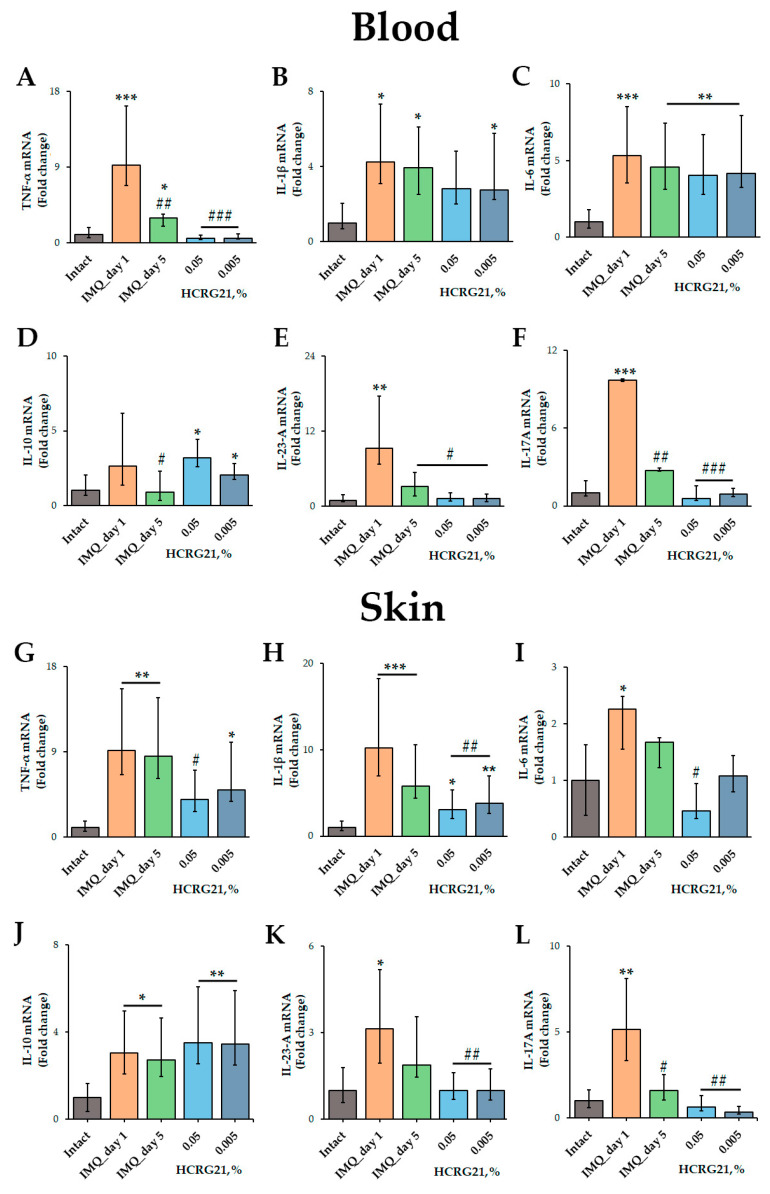
Effects of HCRG21 on psoriasis-related cytokine levels in lesions. Relative blood and skin mRNA expression of TNF-α (**A**,**G**), IL-1β (**B**,**H**), IL-6 (**C**,**I**), IL-10 (**D**,**J**), IL-23-A (**E**,**K**), and IL-17A (**F**,**L**) were quantified on days 1 and 5 of treatment for mice treated with IMQ only and on day 5 of treatment with 0.05 and 0.005% HCRG21 gels. Results are normalized to β-actin gene expression and presented as mean ± SEM (*n* = 7); * *p* < 0.05, ** *p* < 0.01, and *** *p* < 0.001 indicate significant differences from the intact group, and ^#^ *p* < 0.05, ^##^ *p* < 0.01, and ^###^ *p* < 0.001 indicate significant differences from the IMQ-treated group on the first day according to one-way ANOVA/Dunnett’s multiple comparisons tests.

To estimate the effect of HCRG21 on the production of pro-inflammatory cytokines, qPCR and immunoassays were carried out. The qPCR was conducted using mRNA isolated from psoriatic scabs and whole blood samples using gene-specific primers for *Tnf*, *Il1β*, *Il6*, *Il10*, *Il17A*, and *Il23a*. According to the qPCR results, IMQ increased the expression of all cytokine genes tested in both blood and skin cells (Figure 5). We found that 0.05% and 0.005% HCRG21 gels significantly reduced the mRNA levels of TNF-α (*p* < 0.001), IL-23-A (*p* < 0.05), and IL-17A (*p* < 0.001) in the blood compared to the IMQ-treated group on day 1 of treatment (Figure 5A,E,F). The mRNA levels of IL-10 were slightly lower in mice treated with gel containing 0.005% HCRG21, but they were a little higher in the mice treated with the 0.05% HCRG21 gel (Figure 5D). Notably, both HCRG21 gels also slightly decreased the expression levels of the IL-1β gene in the blood, although this did not reach statistical significance (Figure 5B).

In psoriasiform skin, as in blood cells, HCRG21 at both concentrations significantly decreased mRNA levels of IL-1β (*p* < 0.01), IL-23-A (*p* < 0.01), and IL-17A (*p* < 0.01) compared to IMQ-treated skin on day 1 (Figure 5H,K,L), while the 0.05% gel also reduced TNF-α (*p* < 0.05) and IL-6 (*p* < 0.05). It should be noted that both HCRG21 gels equally reduced the mRNA levels of IL-23-A in the skin (Figure 5K), while 0.005% gel inhibited the levels of the IL-17A gene more strongly (Figure 5L).

**Figure 6:**
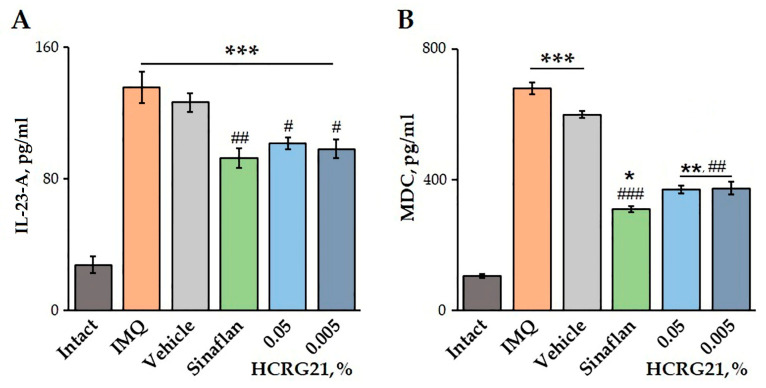
Effects of HCRG21 gels on IL-23-A (**A**) and MDC (**B**) levels in mouse blood plasma evaluated via ELISA. Intact—healthy animals; IMQ—IMQ-treated mice without treatment; Vehicle—mice treated with IMQ and hydrogel without HCRG21; Sinaflan—mice treated with IMQ and Sinaflan; HCRG21 0.05%—mice treated with IMQ and 0.05% HCRG21 gel; HCRG21 0.005%—mice treated with IMQ and 0.005% HCRG21 gel. Results are presented as mean ± SEM (*n* = 7); * *p* < 0.05, ** *p* ˂ 0.01, and *** *p* < 0.001 indicate significant differences from the control group (untreated with IMQ), and ^#^ *p* < 0.05, ^##^ *p* < 0.01, and ^###^ *p* < 0.001 indicate significant differences from the IMQ-treated group according to one-way ANOVA followed by Tukey’s test.

The effects of HCRG21 on blood plasma levels of IL-23-A and MDC were quantified using an ELISA. Application of IMQ was shown to increase the production of both IL-23-A and MDC by 79.6 and 84.5%, respectively, compared to the healthy mice (Figure 6). Treatment of mice with severe psoriasiform lesions with gels containing 0.05 and 0.005% HCRG21 resulted in a significant decrease in IL-23-A levels by 25.2 and 27.5%, as well as MDC levels by 45.7 and 45.0%, respectively, compared to the IMQ group. Notably, the effect of HCRG21 was comparable to the effect of the Sinaflan ointment, reducing blood plasma IL-23-A and MDC levels by 31.7 and 54.4%, respectively (Figure 6).

## 3. Discussion

Psoriasis and ACD are inflammatory skin diseases characterized by epithelial alterations. While psoriasis is associated with possible autoantigens and dysregulated immune responses, ACD is triggered by environmental allergens [29]. Psoriasis is characterized by an abnormal proliferation of keratinocytes and excessive immune cell infiltration in the epidermis and dermis [30,31]. ACD is triggered by re-exposure of haptens in sensitized individuals and involves T cell-mediated epithelial alterations [32].

Psoriasis and ACD treatment is a complex process that aims to relieve flare-ups and extend the period of remission. While a complete cure for psoriasis or ACD is not possible, there are several methods available to help manage the condition [2,6]. Topical therapy, such as creams, ointments, and lotions that contain corticosteroids, vitamin D3 analogues, and salicylic acid, is the most common form of treatment. Physical therapy, including UV phototherapy, can also be used to help reduce symptoms. For more severe cases, systemic therapy with cytostatics and retinoids may be prescribed [33]. New-generation biological agents are being developed to target specific inflammatory processes in the body. The main attention is on inhibitors of various cytokine receptors that reduce the levels of a certain cytokine [11]. However, very little attention has been paid to the search for compounds that directly affect ion channels involved in the pathogenesis of these diseases.

Recently, studies have demonstrated a close relationship between TRPV1 and pathogenesis of both psoriasis and allergic dermatitis [22]. The TRPV1 channel is known to be a primary cellular sensor of thermal and chemical stimulation in the skin tissues, in particular, keratinocytes, peripheral sensory nerve fibers, and immune cells [34]. Overexpression of TRPV1 was found to positively correlate with itching in psoriasis and dermatitis [13]. Increased mRNA expression of TRPV1 was observed in peripheral blood mononuclear cells of patients with psoriasis [35], while knockout of the TRPV1 gene in the IMQ-treated mice resulted in a reduction in inflammatory cell infiltration and the expression levels of inflammatory cytokines (IL-1β, IL-6, IL-23-A, and S100A8) in skin lesions [23]. In addition, a non-vanilloid TRPV1 antagonist, PAC-14028, has been shown to attenuate the inflammatory response and pruritus associated with dermatitis, as well as to prevent barrier damage and accelerate skin barrier recovery [36,37].

It has been shown that psoriasis-like inflammation is triggered by the abnormal activation of specialized immune cells in the skin that produce IL-17 in response to IL-23 [38,39]. Riol-Blanco et al. convincingly demonstrated that TRPV1, through its interaction with dermal dendritic cells (DDCs), regulates the IL-23/IL-17 pathway and controls immune responses in the skin [24]. The genetic or pharmacological ablation of TRPV1 was shown to prevent most IL-23 production by DDCs, while subcutaneous administration of IL-23 restores the inflammatory response.

Here, we first demonstrate the in vivo anti-dermatosis effect of negative modulation of the TRPV1 channel using the peptide HCRG21, which was discovered by us during earlier sea anemone *H. magnifica* transcriptome studies [25]. The recombinant HCRG21 was shown to block 95% of capsaicin-induced current through TRPV1 channels expressed in *Xenopus laevis* oocytes (EC_50_ 6.9 µM). Moreover, HCRG21 had no significant effects on the closed ion channel and did not interfere with the binding of capsazepine, a TRPV1 antagonist [25].

The influence of HCRG21 on ACD was demonstrated in the DNFB-induced murine model. DNFB is known to be the most commonly used hapten for imitation of allergic dermatitis [40]. Its application on mouse ear skin results in pronounced keratinocyte proliferation, strong hypervascularization, immune cell infiltration, and cytokine response [41]. The inflammatory response developing during ACD has been found to be led by allergen-specific T cells and characterized by increased levels of leucocytes and infiltration and/or activation of different immune cell populations, including neutrophils, eosinophils, and mast cells [42,43,44].

Application of gels with 0.05 and 0.1% HCRG21 has been shown to alleviate the clinical manifestations of ACD induced by DNFB, significantly reducing the severity of erythema by decreasing hyperkeratosis and hyperplasia and increasing the rate of epidermis recovery (Figure 1 and Figure 3). The peptide reduced the level of WBCs through the normalization of neutrophil, basophil, and lymphocyte levels, and it slightly decreased the contents of eosinophils, monocytes, and platelets in the blood of DNFB-treated mice (Table 1). This indicates a general anti-inflammatory effect of HCRG21. Notably, platelet levels can become increased during allergic and inflammatory responses, which are often accompanied by blood clotting. Application of glucocorticosteroid-containing ointments like prednisolone and fluocinolone acetonide (Sinaflan) can exhibit similar side effects, one of which is stimulation of thrombocytosis [28,45,46]. Indeed, application of Sinaflan ointment on DNFB-induced mouse skin resulted in an increase in platelet levels, while HCRG21 reduced them. In addition, both HCRG21 gels decreased pro-inflammatory cytokines IL-6 and IL-23-A levels, also confirming their anti-inflammatory effect. According to Riol-Blanco et al., the desensitization of TRPV1^+^ nociceptive sensory neurons by the antagonist resiniferatoxin resulted in an inhibition of IL-23-A production by DDCs and a reduction of the inflammatory reaction in IMQ-induced mice [24]. Based on this, we might suggest that HCRG21 exhibits TRPV1-mediated anti-dermatosis effects.

The influence of HCRG21 on psoriasiform-like lesions was studied using an IMQ-induced mouse model. Prolonged application of IMQ has been shown to recapitulate molecular mechanisms and phenotypic changes in psoriasis. In particular, IMQ causes the production of various proinflammatory cytokines, such as TNF-α [47], IL-1β [48], and IL-23-A [49,50], which drives the activation of Th17 cells producing IL-17A and IL-22 cytokines. In turn, IL-17A activates the IL17 receptor of keratinocytes and triggers the production of several pro-inflammatory cytokines and chemokines (including MDC), recruiting immune cells and directing them to the IMQ-treated tissues. This finally causes hyperproliferation of keratinocytes and the generation of psoriasiform dermatitis [38,51].

In our study, we showed that topical application of gels containing 0.005 and 0.05% HCRG21 attenuated the severity of clinical manifestations of psoriasis, including hyperkeratosis, scaling, and hyperemia, in the affected skin area (Figure 4). HCRG21 possessed general anti-inflammatory effects, significantly reducing the increased levels of WBCs, neuthrophils, eosinophils, basophils, and platelets in mouse blood induced by IMQ (Table 2). In addition, HCRG21 reduced the expression levels of *Tnf*, *Il1β*, *Il6*, *Il23a*, and *Il17a* in both psoriatic scab and blood cells (Figure 5) and inhibited protein production of IL-23-A and MDC levels compared to the values in the IMQ-induced groups (Figure 6), confirming positive anti-psoriasis dynamics. As in the ACD model, inhibition of IL-23-A and consequently IL-17A cytokine production by HCRG21 may be mediated through effects on TRPV1, whose involvement in the suppression of proinflammatory cytokines, in particular Il23a, has been shown by Zhou et al. [23]. Moreover, the peptide significantly increased the expression levels of IL-10 mRNA in both psoriasiform blood and skin (Figure 5D,J). It is notable that IL-10 is an anti-inflammatory cytokine produced by regulatory T cells during inflammatory reaction development, and its production apparently depends on TRPV1 channel state. Zhou et al. have provided evidence that the expression of IL-10 mRNA was increased in IMQ-treated TRPV1 gene knockout mice compared to wild type mice, concluding with the suggestion that TRPV1 is a negative regulator of IL-10 gene expression [23]. Based on these data and our results, we hypothesize that the anti-dermatosis effects of HCRG21 are associated with an upregulation of the TRPV1-mediated IL-23/IL-17 signaling pathway. Despite similar results of proinflammatory cytokine inhibition and hematological parameters, HCRG21 revealed a more prominent repairing effect in a psoriasis-like mouse model than in an ACD-like model, which can be associated with greater involvement of TRPV1 in psoriasis development.

## 4. Materials and Methods

### 4.1. Preparation of Gel with HCRG21

A sample of sodium carboxymethyl cellulose (Na-CMC, Sigma-Aldrich, St. Louis, MO, USA) was dissolved in sterile bi-distilled water to a final concentration of 7% and left to swell at room temperature for 3 h. The hydrogel was heated in a water bath at 80 °C for 1 h, citric acid was added (to a final concentration of 0.7%), and heating was continued for 3 h with periodic stirring. The pH of the hydrogel was normalized with a saturated solution of NaHCO_3_ up to a value of 5.5. An aqueous solution of sodium hyaluronate (2:1, *v*/*v*) or glycerol (3:1, *v*/*v*) was added to the obtained hydrogel.

HCRG21 was obtained via recombinant production, as described in [25]. Briefly, *Escherichia coli* BL21 (DE3) strain cells were transformed by pET32b(+)/*hcrg21* expression plasmid via electroporation using a Multiporator (Eppendorf, Hamburg, Germany) device. The expression of the fusion protein TRX-6His-HCRG21 in BL21 (DE3) cells was induced by an addition of 0.2 mM isopropyl β-d-1-thiogalactopyranoside (IPTG) and cultured at 18 °C for 16–18 h. Fusion protein was purified using a Ni-NTA-agarose (Qiagen, Hilden, Germany) and cleaved by CNBr with a molar ratio to protein of 600:1 in the presence of HCl (up to 0.5 M) for 18 h at RT in the dark, as described in [52]. Target peptide was purified using RP-HPLC on a Jupiter C4 column (10 mm × 250 mm) (Phenomenex, Torrance, CA, USA). Chromatographic separation was performed using a 0–70% gradient of acetonitrile with 0.1% trifluoroacetic acid (TFA, Sigma-Aldrich, St. Louis, MO, USA) over 70 min at a flow rate of 3 mL/min. UV detection was monitored at 214 nm. The purity of the target peptide was verified via mass spectrometry using an Ultraflex III MALDI-TOF/TOF mass spectrometer (Bruker, Bremen, Germany) with a nitrogen laser (Smart Beam, 355 nm), reflector, and potential LIFT tandem modes of operation. Sinapinic acid was used as a matrix.

A weighted portion of the peptide (1 mg) was dissolved in 100 μL of purified distilled water, then dissolved in prepared Na-CMC hydrogels to the final concentrations of 0.005, 0.05, and 0.1%, which were chosen based on the results in [53] and preliminary experiments. The resulting gels were stored at 4 °C.

### 4.2. Animals

This study was conducted on mature (8-week-old) male CD-1 albino mice, weighing 30 ± 2 g. The animals were kept under controlled environmental optimal parameters with a temperature of 23 ± 3 °C, humidity of 50%, and a 12 h lighting cycle. The mice had constant access to balanced Delta feed, laboratory animal feed, and filtered water. All experiments with animals were carried out in accordance with the European Commission’s legislation (Directives 86/609/EEC, 2010/63/EU) “On the protection of animals used for scientific purposes”, guidance on the operation of the Animals (Scientific Procedures) 2000/Act 1986. Animal tests were approved by the local ethics committee of the PIBOC FEB RAS, No. 03/23, from 11 September 2023. Before the experiments, all animals underwent a 14-day adaptation period, after which they were randomly divided into six groups (four control and two experimental) for both ACD and psoriasis-like models. Each group includes seven mice.

### 4.3. Allergic Contact Dermatitis Model

A murine model of ACD in vivo was induced by obligate allergen 2,4-dinitrofluorobenzene (DNFB) (Sigma-Aldrich, St. Louis, MO, USA). CD-1 mice were sensitized to 0.1 mL of 0.5% DNFB in acetone/olive oil (4:1, *v*/*v*), which was topically applied to abdominal skin shaved using an electric clipper. On the 6th and 7th days, 0.02 mL of 0.2% DNFB solution was applied to the dorsal and ventral sides of each ear. HCRG21 was used as part of a hydrogel based on Na-CMC with glycerol. The hydrogel without the peptide was used as the salve control. The treatment was started 24 h after the last DNFB induction. Hydrogels with 0.1% and 0.05% HCRG21, salve control, and the glucocorticoid ointment Sinaflan (fluocinolone acetonide 0.025%, STADA CIS, Moscow, Russia) used as a positive control were applied topically daily for 5 consecutive days. During the application of the drugs, the severity of dermal lesions was evaluated daily via assessment of erythema (hyperemia, lichenification, hemorrhage tissue areas). The erythema level was evaluated visually using a standardized grading scale: 0—absence of erythema; 1—slight erythema (pink skin color); 2—moderate erythema (reddish skin color); 3—marked erythema (red skin color); 4—very marked erythema (red-brown skin color). On the termination day, the blood samples were collected for hematological and immunological assays via cardiac puncture. Ears were excised for histological analysis.

### 4.4. IMQ-Induced Psoriasis-like Skin Damage Model

The back skin of the mice was shaved using an electric clipper, then a depilatory cream was applied to remove residual hairs 1 day before treatment with IMQ. Psoriasis-like skin inflammation was generated via daily topical application of 50 mg of 5% IMQ cream “Keravort” (Glenmark Pharmaceuticals, Mumbai, India) on the shaved back skin for 30 consecutive days to induce the disease. The treatment was started 24 h after the last IMQ induction. HCRG21 was used as part of a hydrogel based on Na-CMC with sodium hyaluronate. Hydrogels with 0.05% and 0.005% HCRG21, salve control (hydrogel without the peptide), and the glucocorticoid ointment Sinaflan (STADA CIS, Moscow, Russia) used as positive control were applied topically daily for 5 consecutive days. During the application of the drugs, the severity of skin lesions was evaluated daily. To evaluate the severity of the skin lesions, a scoring system was used based on PASI (Psoriasis Area Severity Index), which is calculated using the following formula:

PASI = 0.2 × ((R + T + S) × A)(1)

where R is the redness score (0–4); T is the thickness score of plaques (0–4); S is the scaliness score of plaques (0–4); and A is the area of psoriatic involvement score.

On the termination day, whole blood was collected for hematological and immunological assays.

### 4.5. Blood and Tissue Sampling

On the termination day, blood samples were collected from anesthetized mice (intramuscular injection of a mixture of Zoletil (Valdepharm, Val-de-Reuil, France) and Rometar (Bioveta, Ivanovice na Hané, Czech Republic) at 25 and 30 mkg/kg, respectively) via cardiac puncture. Whole blood was collected in tubes with ethylenediaminetetraacetic acid (Improve Medical Instruments, Guangzhou, China). Part of the whole blood was used for RNA isolation and hematological assays, and the rest blood volume was used for ELISA. To separate the plasma, the whole blood was centrifuged at 1000 rpm for 20 min, then blood plasma samples were stored at −80 °C before being used for immunological assays. After blood collection, the mice were sacrificed, and the ears were excised for histological evaluation.

### 4.6. Hematological and Immunological Assays

Whole blood was used for hematological analysis with a Mindray BC-5000 Vet hematology analyzer (Mindray, Shenzhen, China). Cytokine levels were measured in murine blood plasma samples using an enzyme-linked immunosorbent assay (ELISA) diagnostic kit (Cloud-Clone Corp., Wuhan, China) specific to IL-23-A and Macrophage Derived Chemokine (MDC). The ELISA was conducted in accordance with the manufacturer’s instructions.

### 4.7. Histopathological Assays

For histological evaluation, murine ear tissue samples were fixed using a 10% formalin solution (Biovitrum, St. Petersburg, Russia), dehydrated with isopropanol (Biovitrum, St. Petersburg, Russia), embedded in paraffin (Biovitrum, St. Petersburg, Russia), cut into 5 µm-thick sections using a semi-automatic rotary microtome Rothmik 2M (Orion Medic, Moscow, Russia), and transferred onto PCI slides (Citotest, Haimen, China). Deparaffinized ears sections were stained with hematoxylin and eosin (Biovitrum, Russia) and covered with mounting media (Biovitrum, Russia) and a glass coverslip (Citotest, Haimen, China). Ear sections were examined using a Leica DM IL LED light microscope (Leica, Wetzlar, Germany) at 20× magnification. Epidermal thickness was quantified using ImageJ 1.54g software (NIH, Bethesda, MD, USA).

### 4.8. Quantitative Real-Time PCR

Total RNA was isolated from whole blood cells using the Extract RNA reagent (Evrogen, Moscow, Russia) according to the manufacturer’s instructions. Whole blood cells were homogenized in Extract RNA reagent and vortexed, and 1/5 volume of chloroform was added to the supernatant. After incubating the mixture at a room temperature for 5 min, the samples were centrifuged at 12,000 rpm for 15 min at 4 °C. The aqueous phase was transferred to a new microcentrifuge tube, and the same volume of isopropyl alcohol was added. The samples were incubated at room temperature for 10 min and centrifuged at 12,000 rpm for 10 min. The precipitated RNA pellets were washed once with 75% ethyl alcohol, dried at room temperature for 5–7 min, and dissolved in nuclease-free water (Ambion, Austin, TX, USA). RNA purity and quantity were evaluated using a Nanodrop One spectrophotometer (Thermo Scientific, Rockford, IL, USA). The obtained RNA was treated with DNAse I (Thermo Scientific, Rockford, IL, USA) according to the manufacturer’s instructions.

Complementary DNA (cDNA) was synthesized from 1 μg of DNAse I-treated RNA per sample using the MMLV RT kit (Evrogen, Moscow, Russia). The reverse transcription reaction of 1 μg RNA was performed in a 20 μL reaction mixture containing 4 μL of 5× MMLV RT buffer, 2 μL of dNTP mixture (10 mM), 1 μL of MMLV reverse transcriptase (100 U/μL), 2 μL of DTT (20 mM), and 2 μL of oligo dT_15_-primer (20 μM) in nuclease-free water. The reaction was performed at 37 °C for 1 h and 70 °C for 10 min using a DNA Engine Thermal Cycler (Bio-Rad, Philadelphia, PA, USA).

Quantitative PCR (qPCR) was performed using a LightCycler 96 Instrument (Roche, Basel, Switzerland), Biomaster HS-qPCR SYBR Blue (Biolabmix, Novosibirsk, Russia), and gene-specific primers for *Tnf* (NM_013693.3), *Il1β* (NM_008361.4), *Il6* (DQ788722.1), *Il10* (NM_010548.2), *Il17a* (NM_010552.3), *Il23a* (NM_010548.2), and *Actb* (NM_007393.5). The PCR primer sequences are shown in Table 3. PCR was carried out in 20 μL of reaction mixture with 1 μL of cDNA products (1.5 μg/μL) used as templates, as follows: 95 °C for 5 min, followed by 40 cycles of denaturation at 95 °C for 10 s, primer annealing at their specific temperature (Table 3) for 15 s, and elongation at 72 °C for 25 s, followed by fluorescence reading. A melting curve was generated at the end of the reaction to evaluate the specificity of the PCR. The data were analyzed using LightCycler 96 Software Ver. 1.1.0.1320 (Roche, Basel, Switzerland). Quantification of the expression of each target gene was determined using the 2^−ΔΔCT^ method via comparison of the experimental and control groups, where the β-actin gene was used as a reference [54].

### 4.9. Statistics

All data were obtained as three independent replicates, and calculated values were expressed as mean ± standard error of the mean (SEM). One-way analysis of variance (ANOVA) followed by Turkey’s post hoc test was performed to determine statistical significance for all in vivo tests using OriginPro 8.5 software. An ANOVA followed by Dunnett’s multiple comparison test was performed to determine statistical significance for qPCR data using SigmaPlot 14.0 (Systat Software Inc., San Jose, CA, USA). The numbers of repeats, animals (*n*), and statistical tests used are indicated in the figure legends. Differences in the data were considered statistically significant when *p* < 0.05.

## 5. Conclusions

In conclusion, our study provided new insights into the anti-inflammatory effects of HCRG21, a peptide blocker of TRPV1 from sea anemone *H. magnifica*. We first showed that topical application of HCRG21 exhibits therapeutic effects on ACD and psoriasis by inhibiting inflammatory immune responses and relieving clinical manifestations via TRPV1-associated suppression of cytokine gene expression. This provides a target strategy for the treatment of psoriasis and dermatitis in the future. Moreover, HCRG21 exerts protective effects comparable to commercial steroid ointments and may be considered as an alternative non-steroidal compound for dermatological disease therapy, depending on the outcome of clinical trials in humans.

## Figures and Tables

**Table 1:** Hematological parameters in the blood of DNFB-treated mice.

Parameter	Animal Groups					
	Intact	DNFB	Sinaflan	Vehicle	HCRG21	
					0.1%	0.05%
WBC, ×10^9^/L	4.17 ± 0.6	6.36 ± 0.38 *	4.04 ± 1.06 ^#^	6.05 ± 0.77 *	5.42 ± 0.24 ^#^	5.68 ± 0.20 ^#^
NEU, %	26.58 ± 1.62	31.82 ± 3.15	42.32 ± 5.44 ^#^	35.78 ± 1.01	32.40 ± 2.01	34.32 ± 1.29
LYM, %	64.08 ± 1.24	60.42 ± 4.31	48.98 ± 5.21 ^#^	57.38 ± 1.52	59.84 ± 2.28	58.42 ± 1.98
MON, %	5.38 ± 1.03	5.27 ± 1.30	4.47 ± 0.68	3.53 ± 0.34	4.46 ± 0.61	5.38 ± 0.46
EOS, %	3.74 ± 0.44	2.40 ± 0.44	4.22 ± 0.68	3.28 ± 0.56	3.30 ± 0.04	3.68 ± 0.32
BAS, %	0.22 ± 0.07	0.08 ± 0.03	0.02 ± 0.02	0.05 ± 0.03	0.10 ± 0.05	0.24 ± 0.2
RBC, ×10^12^/L	9.46 ± 0.07	9.42 ± 0.10	9.66 ± 0.14	9.35 ± 0.27	9.15 ± 0.26	9.56 ± 0.09
HTC, %	0.49 ± 0.01	0.45 ± 0.01	0.47 ± 0.01	0.45 ± 0.01	0.45 ± 0.01	0.46 ± 0.01
PLT, ×10^9^/L	279.50 ± 73.53	439.00 ± 138.5 *	441 ± 20.17 *	385.33 ± 17.13 *	350.83 ± 0.17 *	350.67 ± 25.31 *
PCT, %	1.94 ± 0.59	2.44 ± 0.74 *	3.10 ± 0.92 *	2.72 ± 0.53 *	2.64 ± 0.26 *	1.20 ± 0.28
HGB, g/L	155.20 ± 2.18	144.75 ± 2.06	149.2 ± 3.34	145.00 ± 3.81	145.80 ± 4.14	149.50 ± 1.26

HGB—hemoglobin; RBC—red blood cells; HTC—hematocrit; PLT—platelets; PCT—plateletcrit; WBC—white blood cells (leucocytes); Leukocyte formula (the white blood cell differential count): LYM—lymphocytes; NEU—neutrophils; MON—monocytes; EOS—eosinophils; BAS—basophils. Intact—healthy animals; DNFB—DNFB induction without treatment; Sinaflan—DNFB induction with Sinaflan treatment; Vehicle—DNFB induction with gel base for HCRG21 treatment; HCRG21 0.1%—DNFB induction with HCRG21 0.1% gel treatment; HCRG21 0.05%—DNFB induction with HCRG21 0.05% gel treatment. Results are presented as mean ± SEM (*n* = 7). The significance of differences was estimated via a one-way ANOVA followed by Tukey’s test versus the control group, with *p* < 0.05 *, and versus DNFB-treated animals, with *p* < 0.05 ^#^.

**Table 2:** Hematological parameters in the blood of IMQ-treated mice.

Parameter	Animal Groups					
	Intact	IMQ	Sinaflan	Vehicle	HCRG21, %	
					0.05	0.005
WBC, ×10^9^/L	3.48 ± 0.18	9.26 ± 1.82 ***	6.30 ± 1.84 **^,##^	8.47 ± 2.12 ***	7.15 ± 0.97 ***^,#^	7.66 ± 1.15 ***^,#^
NEU, %	27.78 ± 1.63	44.67 ± 3.94 **	50.58 ± 4.38 *	43.75 ± 3.53 **	36.05 ± 5.61 *^,#^	31.84 ± 8.02 *^,#^
LYM, %	65.44 ± 1.52	34.16 ± 7.29 ***	29.14 ± 6.59 *^,#^	38.72 ± 6.94 **	45.30 ± 6.85 **^,#^	45.82 ± 9.33
MON, %	3.18 ± 0.20	24.86 ± 4.22 ***	13.38 ± 3.74 **	10.90 ± 5.16 ***	13.75 ± 6.13 ***^,#^	17.90 ± 6.14 ***
EOS, %	3.38 ± 0.60	9.06 ± 1.75 ***	6.82 ± 1.72 **^,#^	6.48 ± 1.89 **	5.38 ± 1.46 *^,##^	4.42 ± 1.03 ^##^
BAS, %	0.17 ± 0.06	0.20 ± 0.04	0.08 ± 0.04^#^	0.15 ± 0.01	0.17 ± 0.02 ^#^	0.02 ± 0.02 ^#^
RBC, ×10^12^/L	9.30 ± 0.19	5.71 ± 0.80 **	7.11 ± 0.81 *^,#^	7.78 ± 0.32 **	6.41 ± 0.44 *	6.93 ± 1.16 *
HTC, %	0.45 ± 0.01	0.34 ± 0.03	0.40 ± 0.04	0.44 ± 0.01	0.37 ± 0.01	0.39 ± 0.03
PLT, ×10^9^/L	397.80 ± 42.15	470.28 ± 51.2 *	470.60 ± 49.75 *	476.66 ± 34.07 *	342.33 ± 10.28 ^##^	357.2 ± 25.59 ^#^
PCT, %	2.44 ± 0.26	1.75 ± 0.68	2.82 ± 0.23	2.53 ± 0.24	1.99 ± 0.26	2.17 ± 0.19
HGB, g/L	148.20 ± 3.8	113.57 ± 7.66 *	131.20 ± 11.94	140.33 ± 2.23 *	127.66 ± 5.69	127.00 ± 8.15

HGB—hemoglobin; RBC—red blood cells; HTC—hematocrit; PLT—platelets; PCT—plateletcrit; WBC—white blood cells; Leukocyte formula (white blood cell differential count): LYM—lymphocytes; NEU—neutrophils; MON—monocytes; EOS—eosinophils; BAS—basophils. Intact—healthy animals; IMQ—IMQ-treated mice; Sinaflan—IMQ and Sinaflan-treated mice; Vehicle—mice treated with IMQ and hydrogel without HCRG21; HCRG21 0.05%—mice treated with IMQ and gel containing 0.05% HCRG21; HCRG21 0.005%—mice treated with IMQ and gel containing 0.005% HCRG21. Results are presented as mean ± SEM (*n* = 7). Significant differences were estimated via a one-way ANOVA followed by Tukey’s test versus the control group, where * *p* < 0.05, ** *p* ˂ 0.01, *** *p* ˂ 0.001, and versus the IMQ group, where ^#^ *p* < 0.05, and ^##^ *p* ˂ 0.01.

**Table 3:** Primer sequences used for detection of cytokine gene expression.

Gene Name	Forward	Reverse	Annealing Temperature, °C	Fragment Length
*Tnf*	5′-GTGGAACTGGCAGAAGA-3′	5′-ACTGATGAGAGGGAGGC-3′	59	192
*Il1β*	5′-AACCTTTGACCTGGGCTGTC-3′	5′-AAGGTCCACGGGAAAGACAC-3′	56	144
*Il6*	5′-ATCCAGTTGCCTTCTTGGGA-3′	5′-GGTCTGTTGGGAGTGGTATCC-3′	55	103
*Il10*	5′-CCCAGGCAGAGAAGCATGG-3′	5′-TCACTCTTCACCTGCTCCACTGC-3′	52	148
*Il17a*	5′-CTCAGACTACCTCAACCGTTCC-3′	5′-TCCCTCCGCATTGACACAG-3′	53	124
*Il23a*	5′-AGCCTGGAACGCACATGC-3′	5′-TGTTGTCCTTGAGTCCTTGTGGGT-3′	53	131
*Actb*	5′-AGGGAAATCGTGCGTGACAT-3′	5′-AACCGCTCGTTGCCAATAGT-3′	52–60	149

## Data Availability

The original contributions presented in this study are included in the article. Further inquiries can be directed to the corresponding author.

## Abbreviations

The following abbreviations are used in this manuscript:

ACD	Allergic contact dermatitis
TRPV1	Transient receptor potential vanilloid-1
IMQ	Imiquimod
DNFB	2,4-dinitrofluorobenzene
PLT	Platelets
WBCs	White blood cells
DDCs	Dermal dendritic cells
